# Children of Patients Undergoing Psychiatric Treatment: An Investigation of Statutory Support Services After Referrals to Child Protection Services

**DOI:** 10.3389/fpsyt.2020.00527

**Published:** 2020-06-19

**Authors:** Anne Ranning, Carsten Hjorthøj, Kamilla B. Jensen, Frank Cloyd Ebsen, Idamarie Leth Svendsen, Anne Amalie Elgaard Thorup, Merete Nordentoft

**Affiliations:** ^1^Copenhagen Research Center for Mental Health—CORE, Mental Health Center Copenhagen, Copenhagen University Hospital, Copenhagen, Denmark; ^2^Section of Epidemiology, Department of Public Health, University of Copenhagen, Copenhagen, Denmark; ^3^Department of Social Work Education, University College Copenhagen, Copenhagen, Denmark; ^4^Child and Adolescent Mental Health Center, University of Copenhagen, Copenhagen, Denmark

**Keywords:** parental psychiatric disorders, early intervention, child protection, psychiatry, statutory intervention

## Abstract

**Aims:**

Preventive interventions for children of parents with mental illness are widely recommended. Mental health services entrust concern for patients’ children by referrals to child protection services. We investigated service coverage for children following referrals.

**Methods:**

Data from referrals regarding 376 children of adult psychiatry patients over 2008–2012 was linked to information from municipal records and Danish national registers. We conducted Cox regression and used Kaplan–Meier curves to show time to intervention and cumulative incidence of any child and family support services with one-year follow-up from referral date.

**Results:**

At follow-up, 32% of children were provided with a child and family support service on average 73.4 days after referral. The most common services were family treatment (18%) and family counseling (11%). A statutory child assessment was conducted for 21% of children. Contents of the referrals suggested that 60% of children experienced adverse home environments and/or acute situations due to parents’ psychiatric illness. Predictors of initiation of support services included a child living alone with the patient, hazard ratio 2.09 (1.41–3.08), the patient being the mother, hazard ratio 1.72 (1.11–2.65), and an adverse home environment presenting an acute situation specified in referral, hazard ratio 1.89 (1.01–3.51).

**Conclusion:**

Our finding that only one third of children receive support after referrals from psychiatry within an average of three months suggests an underserved population of at-risk children. These findings warrant reconsideration of resource allocation and creation of more efficient intervention strategies to protect at-risk children and prevent development of mental illness and adversity.

## Introduction

Psychiatric illness in a parent affects the whole family, particularly when it concerns children dependent on the care of their parents for well-being and healthy development. These children are at increased risk of developing a mental illness due to genetic influences, shared adverse environmental factors with their parents and gene–environment interactions (1–3). Studies have shown that psychiatric symptoms interfere with parenting capacities, while population-based studies from France, Belgium and Denmark demonstrate that 10–40% of children are removed from home and placed in care when parents have psychiatric disorders (4–6).

Randomized, controlled trial results focusing on child-resilience, parenting skills and family functioning have shown family-based interventions to be effective in promoting well-being and preventing mental disorders in children and preventing their unnecessary separations from their parents (7–10). For these reasons, preventive and supportive interventions are widely recommended (7, 11, 12). A recent review in Lancet Psychiatry has presented a mental health prevention strategy and identifies the children of parents with mental illness (COPMI) as a subpopulation, who, owing to their increased risk for mental illness alone, acquire selective primary preventive intervention to shift expected trajectories towards mental illness (13). A high proportion of 7-year-old COPMI already displays sub-clinical manifestations of mental illness or meet the diagnostic criteria thereof, indicating that primary preventive interventions or secondary preventive interventions are warranted (14).

According to the United Nations’ Convention of the Rights of the Child, all the relevant agencies are responsible for children’s welfare (15). These include staff working in adult psychiatry, responsible for notifying local child protection services upon concern for the children of patients. Referrals are sent for a minority of patients, the children of whom staff is concerned due to their knowledge about the patients’ condition and the child’s general circumstances. Thus, the threshold for referrals is high. The referral procedure exists in most countries outside Denmark, including many parts of Europe, the UK, Australasia and the USA. In this intersection of statutory child protection and adult mental health, effective inter-agency communication is vital to determine necessary intervention by responsible authorities.

Scientific documentation of service coverage is lacking internationally where the requirement should be that decision-makers act on adequately-informed grounds when making structural adjustments and securing necessary resource-allocation for service provision. The long-term perspective is to promote children’s well-being while they are growing up, so increasing their resilience and reducing social deprivation and future mental illness cases as children grow into adults (10).

### Aims of the Study

We aim to document the service coverage for children of psychiatric patients following referrals from Mental Health Services to Child Protection Services by investigating the proportion of children receiving a statutory child and family support service, the time between referral and intervention, as well as types of support services within Child Protection Services.

## Methods

### Data Sources

Data on the written referrals concerned the children of patients treated at three mental health hospitals in the Capital Region of Denmark. Approximately 500,000 individuals live in the catchment areas of the three hospitals, which treat patients of all ages, and with all psychiatric diagnoses. All psychiatric hospitals in Denmark are public. All the referrals had been collected in sequence by the Head Social Workers over 2008–2012. This procedure was initiated to keep track of referrals and follow-up on action taken by local child protection services. Referrals contained personal identification numbers of parents and qualitative descriptions on circumstances which caused concern for the children.

The data drawn from the referrals was linked to information obtained from municipal records and Danish population-based registers. Here, data linkage was facilitated by using the unique personal identification number assigned to all live-born children and new residents in Denmark; established in 1968, it is now used across all the relevant registration systems.

The Danish Civil Registration System (CRS) contains dates of birth and data on gender, address and family members living in the same household from January 1st each year (16). Information on statutory child and family support services (SCFS service), and out-of-home placements of children, was then retrieved from Statistics Denmark, with information dating back to 1980. As Statistics Denmark does not provide information on family-based services, this data had to be obtained directly from municipal records. The authors contacted child protection services in the eight municipalities containing the districts within which the three mental health centers belonged. Child protection services were then contacted through a formal letter, formal email and then follow-up emails or telephone calls. Three municipalities accepted participation, three refused and two failed to respond to any request for assistance.

Information on patients’ diagnosis and treatment was obtained from the Psychiatric Central Research Register (PCRR) (17) listing all psychiatric inpatient contact since 1968, in addition to every outpatient and emergency room contact since 1995 (17). The study was approved by the Danish Data Protection Agency, the Danish Health Data Protection Agency, with informed consent waived by the National Scientific Ethical Committee as stipulated in the Data Protection Act (18).

### Study Population

Altogether, 376 children aged 0–17 years were included as the offspring of 218 patients whom, having been admitted as their parents to the respective mental health hospitals, were the object of a referral sent to child protection services in the three participating municipalities. The parents had been inpatients (n = 211) or exclusively outpatients (n = 3) between 2008 and 2012, although information on four parents was missing in the Psychiatric Central Register.

Referrals are only sent concerning the minority of patients in cases where personnel were concerned about the child’s wellbeing, because the patients’ conditions were assumed to interfere with parenting capacities. Crucially, most parents in the study (86%) had previous psychiatric admission.

### Referrals

**Figure 1:**
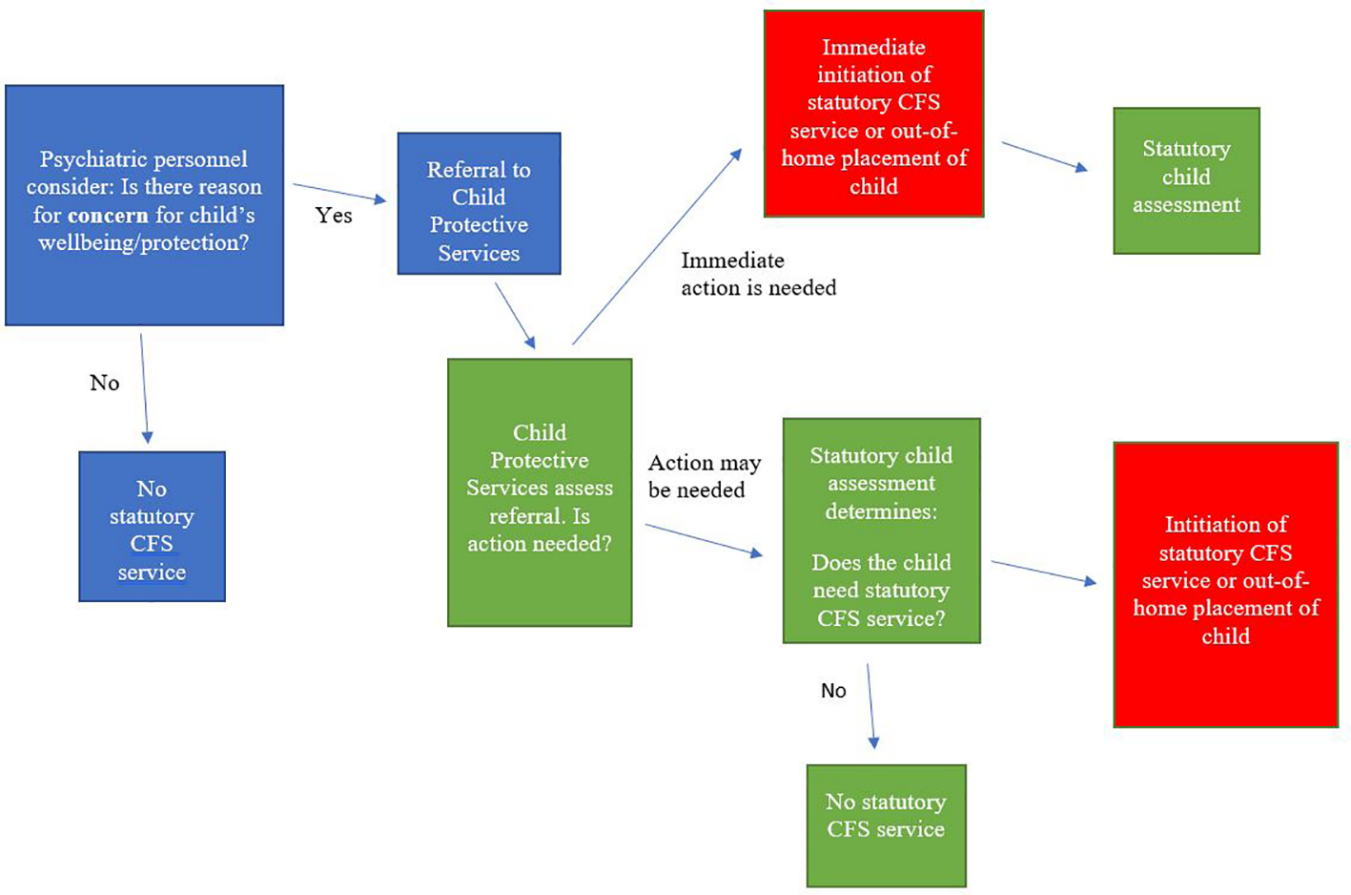
Flow-chart showing the process of referral of children of patients from mental health services to Child Protective Services and subsequent statutory child and family support services (Statutory CFS service).

In cases where a child is or assumed to be in need of special social support, public employees must report their case to the social services department at the local municipality by means of a referral. The way such referrals are handled is shown in **Figure 1**. This form of mandatory reporting is stipulated in section 153 of the Danish Social Services Act (19).

Here, parental consent is unnecessary, although it is preferable where available. It is generally acknowledged that these duty-based referrals override the secrecy duties of health personnel, cf. the Consolidated Health Act (20). Hence, if the child in question is assumed to need special support, a more thorough assessment, a statutory child assessment, must be carried out within 4 months. In cases of acute protection concern for children the assessment can be conducted at the same time of a support service or out-of-home placement.

There are no formal requirements regarding the content of the referrals. For the purpose of this study the researchers classified the referrals according to their contents. The rating was conducted by a child psychiatrist (AT), clinical psychologist (AR) or a research anthropologist (KBJ). Interrater reliability based on 30 cases showed an interclass correlation of 0.87. A referral was classified as a *Vague Concern* when concern was expressed for the child’s wellbeing based on vague not specific grounds. A referral was classified as a *Specific Concern* in presence of specific examples of neglect, abuse and other adverse conditions of upbringing. A referral was classified as an *Acute Concern* when acute situations caused by patients' psychiatric illness and involving children were mentioned in the referral. Vague and Specific Concern were mutually exclusive, while referrals mentioning a critical situation, but not prolonged adverse conditions of upbringing were classified as both a vague and acute concern (see **Appendix 1** for classification criteria). The length and degree of details varied greatly between referrals, some counting several detailed pages and others short providing only the most basic information about the parent’s diagnosis, time of hospitalisation and child’s age.

### Diagnostic Categories of Parents

Information on the parents’ psychiatric diagnoses was drawn from the Danish Psychiatric Central Register. Based on the hierarchy of ICD-10 the following diagnostic hierarchy was used if the parents had more than one diagnosis: The highest up the scale was schizophrenia (F20), then other psychosis (F21–F29), bipolar disorder (F30–31), unipolar depression (F32–34), while the lowest in the hierarchy were other disorders. Substance abuse (F10–F19) and suicide attempt (X60–X84) were considered co-morbid diagnoses.

### Outcome Measures

After the referral date, the primary outcome was initiation and time to initiation of any statutory child and family support service or out-of-home placement by child protection services subdivided into the following categories: 1) Family treatment, 2) Short family counseling/short assessment, 3) Support persons, including family support workers. 4) Financial support including free daycare, 5) Institutional or family-based relief care and 6) Out-of-home placement of child. As a secondary outcome we investigated whether the child had undergone a statutory child assessment i.e. an investigation of the circumstances regarding the child’s family, school and general health etc., to determine the need for statutory intervention.

### Statistical Analysis

Cox regression was conducted to calculate hazard ratios when initializing any statutory child and family support (CFS) service in the first year after referral.

Hazard ratios were calculated as a function of the child’s gender and age group, parent’s gender, parent’s psychiatric diagnosis, classification of referral, child’s living situation, his or her municipality, and whether he or she had previously received a statutory CFS service.

Kaplan–Meier curves were used to determine the initiation of statutory CFS service with the days since referral as the underlying time variable. This analysis was performed in Stata/MP version 16.1. With log-rank test it was analyzed whether there were significant differences in the time lag and the proportion receiving services after referral for children referred for the first time versus children for whom support services were already established at the referral date, as their probability of receiving a new service may differ from children with no previous support service.

## Results

**Table 1** summarizes the characteristics of the 376 children studied. In 31 cases (14.2%), the referral was made upon the parents’ first psychiatric contact while 187 (86%) had previous psychiatric admissions. The median number of previous psychiatric contacts was six. A total of 42 children (11.2%) had already received support from child protection services before each referral date. Referrals concerning 60% of children were classified as either specific or acute concern, or a combination hereof.

**Table 1:** Descriptive characteristics of the cohort of the 376 children of psychiatric patients referred to Child Protection Services by Adult Psychiatric Services.

Characteristics	n/%	Offered service^*^ 1 year following notification in %
Female child	180 (48.6%)	35.0%
Male child	190 (51.4%)	31.8%
Age average	7.9 (SD 5.3)	N/A
Mother is a patient	257 (68.4%)	37.4%
Father is a patient	111 (29.5%)	23.6%
Municipality A	79 (21.0%)	43.0%
Municipality B	266 (70.7%)	31.6%
Municipality C	31 (8.2%)	24.2%
**Parent’s diagnosis**		
Schizophrenia	60 (16.0%)	40.0%
Other psychosis	42 (11.2%)	31.0%
Bipolar disorder	37 (9.8%)	37.8%
Unipolar depression	102 (27.1%)	28.4%
Other mental illness	135 (35.9%)	33.5%
Suicide attempt	8 (2.1%)	50.0%
Substance abuse	67 (17.8%)	33.5%
**Child’s living situation**		
With both parents	137 (36.6%)	27.0%
With the patient	141 (37.7%)	45.5%
With the other parent	75 (20.1%)	25.5%
With neither parent	5 (1.3%)	20.0%
Missing information	16 (4.3)	19.6%
**Children with service at date of referral (Previous support service)**	42 (11.2%)	38.2%
**Classification of referral**		
Vague concern	132 (40.5%)	26.5%
Specific concern	129 (39.6%)	37.6%
Acute concern	26 (8.0%)	30.8%
Vague concern and acute concern	6 (1.8%)	0%
Specific concern and acute concern	33 (10.1%)	42.6%

*Statutory child and family support service and out-of-home placements.

**Figure 2:**
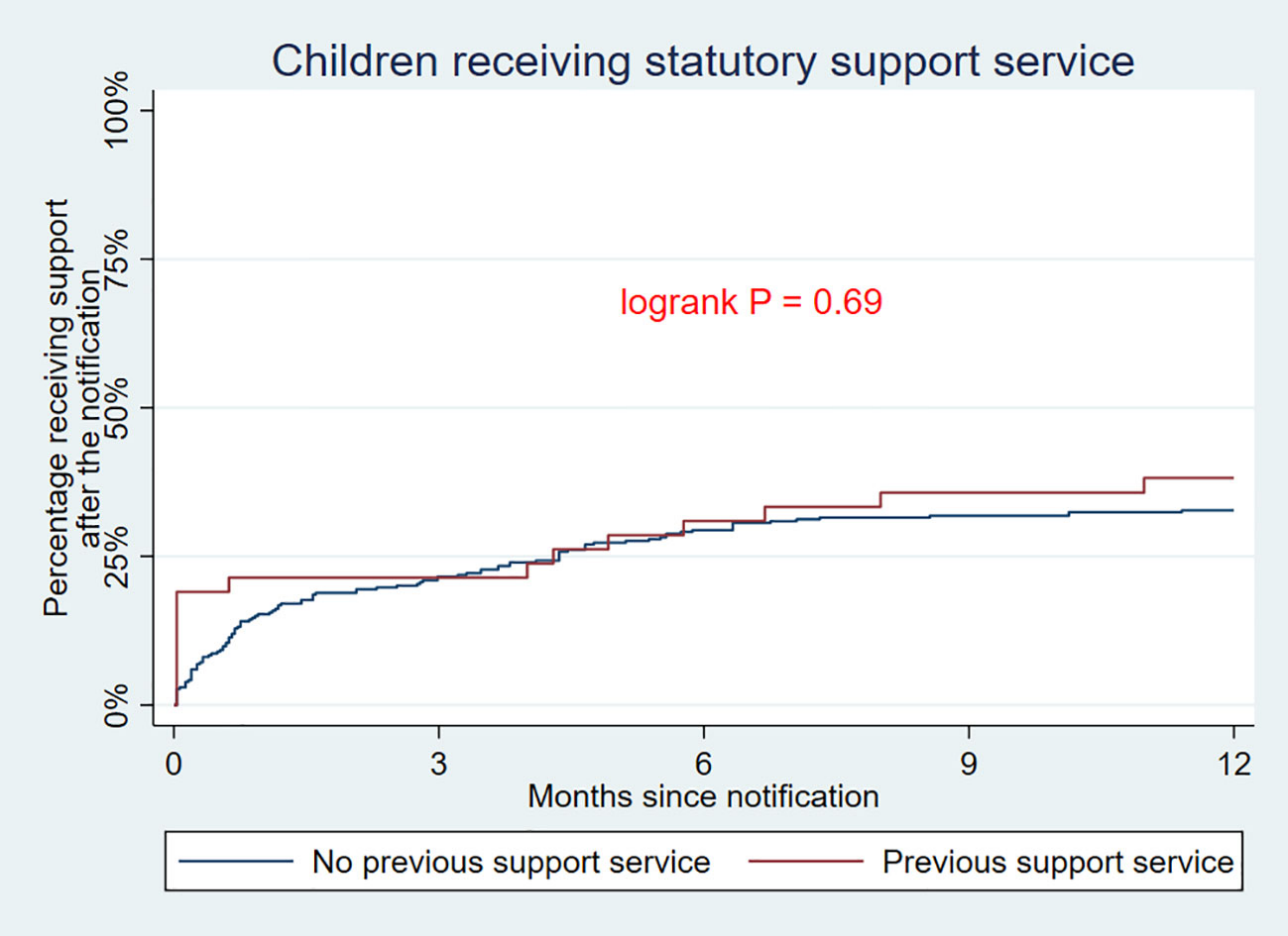
Kaplan–Meier curves depicting the cumulative incidence of any statutory child and family support service or outplacement from referral date.

The Kaplan–Meier curves in **Figure 2** depict the cumulative incidence of any statutory CFS service or outplacement. Here, 32% of children without service at the date of referral obtained a statutory CFS service during follow-up within an average of 73.4 days. For the children receiving an ongoing service at the referral date, 38% obtained a new service during follow-up within an average of 86.8 days. Log-rank test showed no statistical difference between the two groups (**Figure 2**).

**Figure 3:**
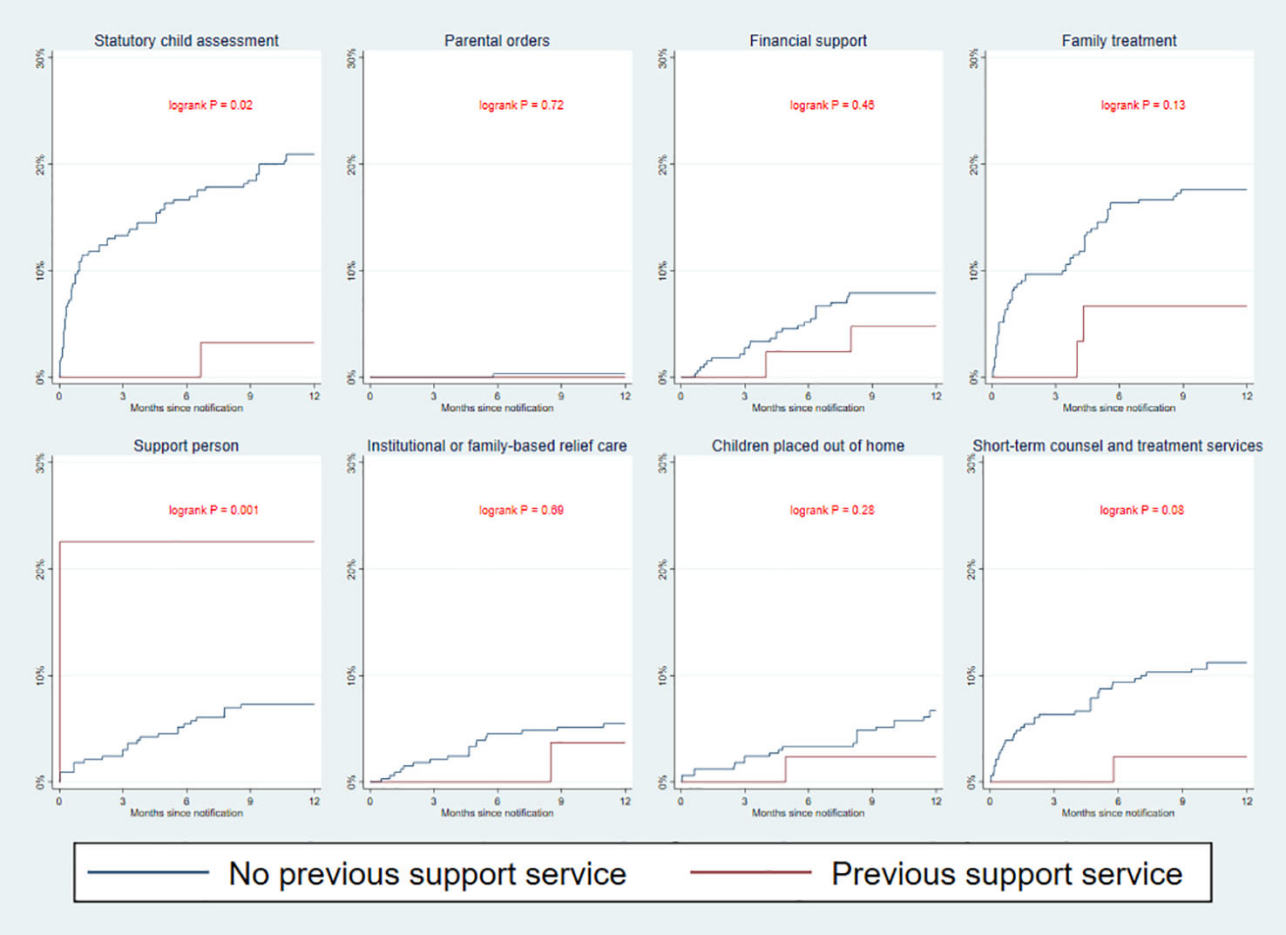
Kaplan–Meier curves depicting the cumulative incidence of specific statutory child and family support service or outplacement from referral date.

The curves in **Figure 3** illustrate specific types of services initiated within a year after referral. When examining the group of children without service at referral, family treatment (18%), short family counseling/short assessment (11%) and financial support (8%) were found to be initialized most frequently. Six percent of children were placed in out-of-home care while 21% of children had been subject to a statutory child assessment at the end of follow-up. Parental orders occurred in only one case, i.e. initiation of child protection services without parental consent. Log-rank test showed statistical difference between the two groups with higher incidence of support persons being appointed to children with prior services compared to those without, and higher incidence of statutory child assessments being initiated for children with no prior services (**Figure 3**).

In recognition that statutory child assessment is a part of the process of service initiation, we performed an explorative analysis of the beginning of child assessment in the support services. For the children with no previous statutory CFS service we found services were provided to 36% of children during follow-up within an average of 79.1 days.

**Table 2** shows the results of the Cox-regression analysis of different predictors of service provision: Compared to children living with both parents, children living alone with a parent in treatment had a significantly higher probability of a service being initialized with a hazard ratio (HR) of 2.09 (1.41–3.08) p value = 0.0002. Classification of both specific concern and acute concern in the same referral was associated with a higher HR of service provision at 1.89 (1.01–3.51), with a p value = 0.05 when a vague concern was the reference. When the mother was the patient, the HR was higher (1.72 (1.11–2.65), p = 0.01) than for fathers. Other factors such as the municipality’s status, parent’s psychiatric diagnosis and child’s gender and age group were not found to be predictive of the initiation of service.

**Table 2:** Hazard ratio of being provided with a statutory child and family support service by the first year as a function of child- and parent-related characteristics.

Characteristics	Hazard ratio (95%CI)	P-value
Female child	1.14 (0.80–1.63)	0.46
Mother is a patient	1.72 (1.11–2.65)	0.01
Father is a patient	1 (ref.)	
Age group 0–5 years	1.11 (0.72–1.72)	0.34
Age group 5–12 years	0.82 (0.51–1.30)	
Age group 13–17 years	1 (ref.)	
Municipality A	1 (ref.)	0.06
Municipality B	0.67 (0.45–1.00)	
Municipality C	0.45 (0.20–1.02)	
**Parents’ diagnosis**		
Schizophrenia	1.57 (0.92–2.70)	0.56
Other psychosis	1.08 (0.56–2.08)	
Bipolar disorder	1.33 (0.70–2.52)	
Unipolar depression	1 (ref.)	
Other psychiatric disorder	1.26 (0.79–2.02)	
Substance abuse	0.98 (0.62–1.56)	0.94
Suicide attempt	1.73 (0.64–4.69)	0.28
**Child’s living situation**		
With both parents	1 (ref.)	0.003
With the patient	1.95 (1.30–2.92)	
With the other parent	0.96 (0.55–1.67)	
With neither parent	0.79 (0.11–5.79)	
**Children with service at date of referral**	1.23 (0.73–2.08)	0.44
**Classification of referral**		
Vague concern	1 (ref.)	0.05
Specific concern	1.44 (0.93–2.23)	
Acute concern	1.14 (0.53–2.46)	
Vague concern and acute concern	No cases of support service	
Specific concern and acute concern	1.89 (1.01–3.51)	

^*^Statutory child and family support service and out-of-home placements.

## Discussion

The study of 376 children mostly involved parents who were repeatedly admitted in-patients, undergoing treatment for psychiatric disorders. The findings were that only one-third of children were provided with a statutory child and family support service following referrals from psychiatric services due to protection concerns for children. For 60% of these children, the referrals specified the existence either of adverse home environments, including child abuse and neglect, and/or critical situations arising due to a parent’s psychiatric disorder. For the children who have received a support service, the mean number of days from referral to intervention was 74. Meanwhile, the children of maternal patients and children who lived alone with the patient had the greatest likelihood of receiving a support service. The children whose referrals included specific examples of poor conditions and acute situations related to their parent’s psychiatric disorder had a better chance of receiving child protection services.

### Possible Mechanisms and Explanations for the Findings

#### Social Services Considered the Referrals to be Unfounded

One possible explanation to the low service coverage after referrals may be that Child Protection Services (CPS) determined the children’s environment not be detrimental and their well-being as unproblematic, thus finding the reasons for concern raised by psychiatric services invalid. However, we find this explanation to be unlikely for most children for several reasons; one being that staff in psychiatric services send referrals only for a selected group of children for whom they are especially concerned, hence a such referral is an indicator of severity in terms of adverse environmental conditions. As recommended by Arango and colleges all children of parents with mental illness (COPMI) should receive selected preventive interventions owing to their high-risk status, and especially those children experiencing multiple environmental risk-factors (13). As 60% of referrals specified that children experienced neglect, abuse or other types of damaging domestic conditions the low level of service coverage is unsatisfactory. Furthermore, a high proportion of COPMI show sub-clinical manifestations of mental illness or meet the diagnostic criteria thereof, indicating that primary- or secondary preventive interventions are warranted (14). As CPS had only conducted a thorough child assessment in 20% of cases, their understanding of children’s environmental conditions are limited, and children with early clinical manifestations or an already-developed mental illness are easily overlooked.

#### Poor Information and Limited Resources

A higher degree of service provision was found associated with referrals for both specific and acute concerns compared to vaguely worded referrals. The existence of imprecise information has indeed been shown to makes it difficult to gauge the degree of urgency and may be associated with longer time delays from referral to accommodation (21). The limited resources available for processing and evaluating the referrals by CPS are a possible explanation of the subsequent low service coverage and long time lag to intervention from referral date. Inadequate resources impede every aspect of social, welfare and health care, while heavy caseloads prevent social workers providing services for these children (22). A likely consequence of both imprecise information in referrals and limited resources is that CPS reacts only to the most serious or acute cases. An average of 73 days was found from referral date until intervention starts. This is a considerable amount of waiting time, yet, it is presupposed in the statutory demands for thorough, holistic assessment of up to 4 months duration. However, our exploratory analyses, did not suggest the process of statutory child assessment to account for the time to service initiation, nor for the proportion of children being accommodated with a service.

#### Legal and Psychological Barriers

A legal barrier for intervention may be that the Consolidation Act of Social Services is based on voluntary participation of the families; hence, parents may reject interventions causing child protection services to close the case. In cases where consent from parents is missing, municipalities can use parental orders, although this approach had only been used in one case in this study. Stigma of mental illness may be a barrier for service provision when a parent has mental illness: Both for parents who experience a clear need for support, but fear disclosing their parenting difficulties out of concern for losing custody of their children (23) and for children who keep silent about home problems because they feel ashamed and fear being placed out-of-home (24).

### Perspectives on Supportive and Preventive Interventions

We found higher incidence for service provision for children of maternal versus paternal patients, as well as for children living with the parent with a psychiatric disorder. This is in accordance with previous register-based studies showing a substantial proportion of COPMI living with a single mother (25) and higher incidence of intervention by Child Protective Services in terms of child-placements in presence of maternal versus paternal psychiatric disorders (5). One potentially fruitful strategy may be to build up services for COPMI within the mental health sector. Even though adult psychiatric services do not traditionally offer support to patients’ children, the existing formal structure does not exclude such services. On the contrary, the responsibilities of the Convention on the Rights of the Child refer to all public authorities. By combining family intervention with the parents’ psychiatric treatment, patients and clinicians with mental health expertise can focus on patients’ recovery while taking care of children’s well-being, with less risk of delay or missing out on possible intervention because of sole reliance on referring the family to social services. This model has been implemented on a national level in Sweden and show improvements in parent–child relationships and child wellbeing (26, 27). The same approach has been applied in Finland and shows reduction in children’s emotional symptoms and anxiety (28, 29). Interventions of these kinds are of low-to-moderate cost and progressive in focusing on stronger functioning families and fostering resilience in children. Improving the possibilities—and obligations—of mental health services to offer support to COPMI would hence be in line with policy trends in other areas, such as school and family law sectors, where counselling and treatment services for at-risk children have been implemented over recent years (30, 31). Another strategy would be more radical change to the infrastructure of the referral process such as the newly developed Finnish “Let’s Talk about Children Service Model (LT-SM)” (32). Here, referrals concerning at-risk children are sent to a “one contact service” connecting relevant stakeholders such as mental health—and social services, kindergartens and schools who join together on a case-based collaboration around the family. Results of the study show that this interagency collaboration is indeed feasible.

### Strengths and Limitations

To the best of our knowledge, the present study is the first to investigate statutory child and family support services for children following referrals of concern from adult mental health services. However, the study has some limitations, one being that we did not have information about referrals from other agencies, such as the child’s school, family doctor, neighbours or others, being sent during the study period. Thus, the causal relation between referrals from mental health services and the delivery of subsequent services is unclear. Although we obtained information on outplacements and relief stays of children, shorter, informal relief stays of children within the social network may have been arranged but not registered in municipal records. In addition, parents may have obtained practical support in the home from the adult service department. Another limitation is that some children may have moved to a different municipality after the date of referral and services have not been initialised for this reason. Such limitations may cause underestimation of the proportion of children accommodated via a service. Another further limitation is then possible selection bias in the municipalities who have either accepted or refused participation in the study. For instance, one municipality refused participation out of concern for public criticism if the proportion of children receiving support was found to be very low. After the 2008–2012 study period, the number of referrals concerning at-risk children increased considerably, with a 20% national increase over 2015–2017 (33). Thus, the proportion of children being accommodated with a support services following a referral has likely decreased in recent years, as the resources of social services have not increased according to demands. Furthermore, it is uncertain to what degree these research findings can be generalized to other countries, with their different organisation of sectors and distribution of target groups. Referrals may be ‘false-negative’ regarding child abuse or neglect, due to a lack of awareness from mental health personnel. The proportion of false-negatives is therefore unknown.

### Concluding Remark

Our findings strongly suggest an under-served population of children of patients with severe psychiatric disorders with severe flaws in the inter-agency organization of entrustment and intervention for a population of vulnerable children and their families. The creation of more effective intervention strategies and better allocation of resources is thus required, with the aim of strengthening resilience and preventing trajectories towards mental illness and adversity for the children concerned.

## Data Availability Statement

The datasets generated for this study will not be made publicly available due to ethical, legal, and privacy restrictions. Requests to access the datasets should be directed to the corresponding author.

## Ethics Statement

The study was approved by the Danish Data Protection Agency, the Danish Health Data Protection Agency, with written informed consent waived from participants waived by the National Scientific Ethical Committee as stipulated in the Data Protection Act (The Ministry of Justice 2018). Ethical approval was not required as per local legislation and national guidelines.

## Author Contributions

All authors contributed to the article and approved the submitted version. CH and AR have been responsible for data management and quantitative analyses. AR, AT, and KJ have conducted the evaluation of contents of the written referrals.

## Funding

The project is supported by Trygfonden. The funding source had no influence on the following: the design and conduct of the study; the collection, management, analysis and interpretation of the data; preparation, review or approval of the manuscript; and the decision to submit the manuscript for publication.

## Conflict of Interest

The authors declare that the research was conducted in the absence of any commercial or financial relationships that could be construed as a potential conflict of interest.

## Appendix 1 Criteria for Classification of the Referrals of Children of Psychiatric Patients

**Table:**

Classification	Contents of the referral
Vague concern:	Expression of concern for the child’s wellbeing based on vague not specific grounds.
Specific concern:	Includes specific examples of neglect, abuse and other detrimental conditions, usually characterized as childhood trauma or prolonged psychological strain affecting children in view of their parents’ psychiatric illness. Neglect was defined as long-term lack of parental attention and nurturing, while child abuse includes psychological and physical abuse. Parental suicide attempts and long-term, daily substance abuse are also rated here.
Acute concern	Mentions acute situations, like those involving the police or when a parental suicide attempt is discovered by the child. The referrals call for authorities to conduct an immediate examination of the child’s life circumstances; an example of an acute concern might be a patient with severe psychotic symptoms walking in the middle of the highway with his 2-year-old child.
